# Step-by-step Guide to Create Competency-Based Assignments as an Alternative for Traditional Summative Assessment

**DOI:** 10.15694/mep.2020.000120.1

**Published:** 2020-06-08

**Authors:** Hebat Allah Amin, Mohamed H. K. Shehata, Samar A. Ahmed

**Affiliations:** 1 Helwan University; 2 Department of Family and Community Medicine; 3 ASU-MENA-FRI-Ain Shams University

**Keywords:** Assignment, Assessment, Student peer assessment, Self-assessment, Pandemic.

## Abstract

This article was migrated. The article was marked as recommended.

The sudden, prolonged COVID-19 lockdown has offered a great challenge to the medical school. This was not only at the level of learning and curricular design but also the level of assessment. The traditional summative assessment tools have collapsed during this Pandemic.

Herein, we provide a five-step guide for designing competency-based E-assignments for summative assessment. Innovative assignments designs are crucially required for fair summative assessment of the medical students, mainly in the pre-clerkship phase. These need to be innovative, engaging, competency-based, well-designed, with defined rubrics, integrated, and interdisciplinary whenever possible.

These should also enforce the concepts of self-assessment and student peer assessment. Including the students in the formulation and design enhances their self-motivation where there is no face-to-face education. Designing an assignment with a quality product as an outcome increases the students’ enthusiasm and self-confidence. A brief case-study is included as an example.

Teaching after the pandemic era will greatly change with inevitable changes in the dogmatic concepts. Formative and summative assessments are probably changing seats which might be sustained for some time post-COVID-19.

## Introduction

There is a generally acknowledged concept that assessment drives learning. This mandate acquiring a new vision for creating assessment tools to meet the shift of the medical curricula to integrated, competency-based learning ( Sohrmann *et al.*, 2020).

Competency-based medical education (CBME), requires an innovative integrated assessment tool. CBME assessment necessitates continuous, criterion-based, work-based strategies. “Best practices” should be highlighted and encouraged. CBME assessment should enhance life-long learning as the expertise is the goal, not merely the competence ( Holmboe *et al.*, 2010).

The new assessment strategy should be tailored in tandem with the development of the new curricula. This could be achieved by strategic planning, including the educators, administrators, and the student to formulate a structured plan for the assignment design ( Ferris, 2015).

A well-designed assignment can guide students through engaging deep learning experiences and divert their attention from grades towards creativity and critical thinking ( Liu and Carless, 2006). Both summative and formative assessment methods are useful when applied in the correct setting and at an appropriate stage of learning. Online problem-based learning techniques have proved to be incredibly popular ( Ahmed *et al.,* 2020).

Novel assessment methods including self and peer assessment strategies enhance students’ engagement and self-motivation. Besides, it promotes developing their critical thinking skills ( Bloxham and West, 2004).

As a result of the COVID-19 lockdown, the Egyptian Ministry of Higher education mandates that medical school should consider student assignments as an alternative for summative assessment for all students in the pre-clerkship phase. This article tries to put a general stepwise approach to plan such assignments where formative and summative assessments are probably changing seats which might be sustained for some time post-COVID-19.

## Steps of conducting a competency-based integrated assignment

### Step one: Position Your Assignment in the Curriculum

#### Identify competency/ competencies to assess

When starting to consider designing an assignment as alternatives for summative assessment object in the curriculum it is advised to take a step back and look to the bigger picture. Revise your national/school’s competency framework program competencies and identify the competencies you will be testing ( Humphrey-Murto *et al.,* 2017). Consider your school’s educational strategy. Assignment in a school that adopts a clinical presentation curriculum would be different from assignments in a school that adopts problem-based learning for example. One more important aspect to consider is the availability of inter-disciplinary cooperation with other institutes, schools, or bodies as this will provide the designed assignments with a new dimension. It is important to have clear objectives for the designed assignments as clearly articulated learning goals and objectives are an important part of any learning activity (Gagne *et al.* 1992; Al-Eraky, 2012).

E-assignments provide an excellent opportunity to encourage students to collaborate together to achieve projects that will help them develop some competencies that are not-uncommonly ignored or minimized during traditional teaching where different departments usually compete for a space in the teaching schedule in integrated medical schools.

#### List basic skills required to be incorporated in the assignment

Place the expected skills to be tested on a comprehensive list. Consider each skill independently and ensure that this is consistent with your complex assessment system considering summative as well as developmental assessments. The electronic assignments triangulate with other methods of assessment and will probably add to the validity, reliability, and most importantly the educational impact of your assessment system. To achieve this, planners might need to map new skills on their assessment blueprint.

#### Revise each skill and align with the competency selected

The skills are aligned to the competencies -The graduate as a health care provider, health care promoter, professional, scholar and scientist, member of the health team, and a part of the lifelong learner and researcher- through mapping a program matrix.

### Step two: Design

Designing an assignment through a holistic visionary strategy can magnify the outcomes. Apply the steps of strategic planning through clarifying the vision, needs assessment, formulation of the assignment/project, implementation, followed by evaluation of the process, and correction actions. This should be established throughout the following steps:

### Form a team

A team representing relevant departments with student’s representation will probably come out with a design that is more applicable and acceptable to various stakeholders.

#### Review of literature

After coming up with initial ideas for assignments’ formats, team members might need to look for similar ideas that were implemented in other medical education institutes to learn from the published experience of other educators.

#### Brainstorm

Allow sub-teams to brainstorm possible formats of assignments creatively. In this initial planning phase, team members should adhere to the Problem-solving process and avoid criticizing the first versions of ideas. In brainstorming several decisions are made and many questions are raised

#### 1. Decisions

##### Degree of integration

Integrative Learning is an approach to education that highlights the importance of addressing real-world issues relevant to students’ life experiences and interests. Hence, Integrative assignments focus on:

• The utilization of multiple modes of inquiry and multiple venues of knowledge
• The application of theory to practice employing interdisciplinary diverse perspectives
• The contextualization of students’ personal experiences in larger societal and global patterns
• We believe that Integrative Learning is essential for students’ success, self, and social responsibility and civic engagement in a rapidly changing and connecting the world ( Sites.google.com, 2020).

##### Format

The format of the assignment should be discussed whether it will be a web-based assignment or a paper form one.

##### Tools

Tools available for executing the assignment should be discussed. It is advised to reflect on the value of the choice of the tools and whether it feeds into the competencies assessed through the assignment. If this is not the case, then the tools used are better left to the student creativity. This will allow a degree of freedom for the students to take ownership of the process and the outcome.

##### Group/individual

Well-designed group assignments with clear, defined, individual roles could be fairly assessed in addition to covering the competency area of teamwork. When considering group-assignments it is important to identify the role of every individual in the team. Possible roles in a group could also be the role of a peer mentor identified from a previous batch to help align the work of the students towards the objective.

##### Purpose

The basic aim is to have a fair and valid effective assessment tool. However, designing the assignment to be engaging and usable magnifies the expected outcomes. The purpose of the assignment and its contribution to the hidden aspect of the curriculum should be discussed elaborately in the brainstorming phase. This will later reflect on all decisions made regarding tools, context, format, etc. This will also reflect on the degree of flexibility the students are given in the assignment.

##### Type of assignment

**Figure 1.**
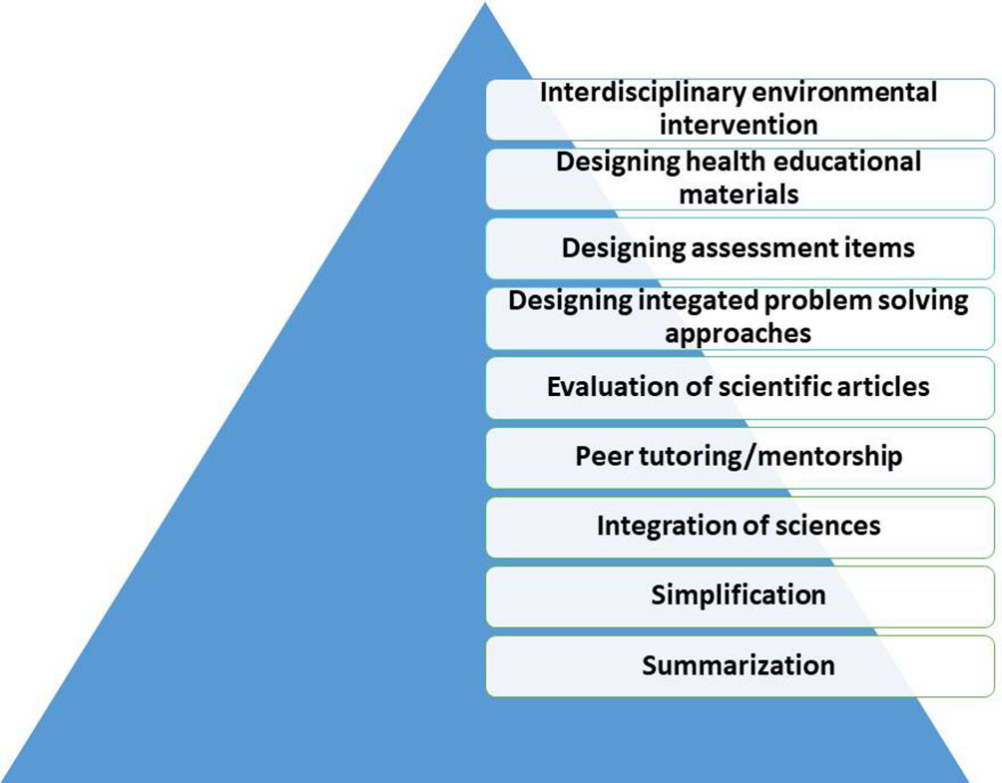
A suggested model of E-Assignments levels

Based on their purpose, assignments designed within a competency-based framework can add value to the learning experience of students. As the team designs the assignments, it is valuable to consider the level of the assignment. Whether they will be only at the summarization level or be at higher levels such as simplification and delivery of information. Whether the assignments include only medical students or provide them with the opportunity to work with a multidisciplinary team is another point to consider. The drive-in this stage will be the usability of the assignments which would be a major motivator for students and staff to create and innovate. In the following diagram ( Figure 1), a model suggested by the authors that describe various levels and examples of E-assignments that can be used.

#### 2. Questions

It is important to spend some time deliberating on a few questions to reflect on the decisions of design. A guide to these questions is offered below.

##### Is the format relevant to the student’s professional future?

The assignment should respect the required competencies and poses an integrated vision between the different disciplines. The context in which the assignment is delivered is an important part of the learning. Creating assignments that respect the future profession, creates better student engagement and reflects later on their life-long learning skills. Students identify learning goals easier when the assignment represents their future profession. An example of this in medical curricula could be assignments that require the student to mimic a doctor-patient encounter in a video format.

##### Is the requirement comparable to the grades/ stake?

The time the student is requested to spend on the assignment should be weighed against the grade assigned to the task or the stake associated with it. An example of this is the time allocated for summative assessment assignments and the degree of complexity associated with it. When assignments are used for differentiating purposes, it is expected that the time the student dedicates for the assignment and the degree of assignment complexity is high.

When learners are encountering a complex topic or if they are unfamiliar with a genre or learning format, provide less material, easier tasks, or more time to complete the assignment. Similarly, learners will be able to cover less material on their own than can be covered in more didactic and interactive sessions like lectures. Independent learning assignments may require more time or less material than typically allotted to a lecture or cover less material than if the same topic were to be covered in a lecture ( Rachul *et al*. 2020).

##### Is there an output/product?

A usable quality product enhances students’ engagement and enthusiasm. Being participants in the formulation of vision magnifies the expected outcome.

##### How can we make the output usable and sustainable?

This could be achieved through having a holistic vision with a clear, challenging, yet applicable goal. To ensure that the assignment product is usable make sure it results in bridging an already existing gap. This can be attained by visiting the course report from previous years and the results of student feedback to identify learning gaps and gaps in resources. When these have been identified they can be then designed into a needed resource list. Given these resources are generated from actual learning gaps they will later be usable for future instruction.

To keep the outcomes and products usable and sustainable, it is recommended that they are hosted on a visible portal where students themselves take the quest to ensure they are publicized to others. Using YouTube where other students can log in their feedback and questions in the future will be very useful as well as using the existing blackboard to host the material for future student reference.

##### Is the requirement quantifiable/measurable?

The clear quantifiable requirement ensures a fair assessment tool.

##### Is there a degree of individuality in the task to protect from designed-in plagiarism?

Balancing out assignments to ensure that different teams are subsequently engaged in the design process is extremely important to ensure the reliability of the process as an alternative for conventional assessment. In our experience when students are asked to design a product, it is an important step to enhance student creativity and thus ensure that students exert the required effort.

Another important step to designing the assignment to prevent plagiarism is to make sure that products of assignments are published after peer review. This means that assignments that produce written content are directed to journal publications and those that produce video content are directed to YouTube publishing. This makes products visible rather than having products of assignments seen solely for teacher evaluation.

##### What are the elements of the product? Can it be broken down?

Designing an integrated, multidisciplinary assignment through a holistic vision can create an enthusiastic project with main and subsidiary outputs. These outputs can include the production of quality educational material adding value to the curricular content. These can also include awareness sessions or videos serving the community inside the campus and even the larger community. Involvement in scholarly research work documenting the educational practices.

Break down these outputs into specific elements. Map each element on the first column of the matrix against the skills listed. Map the elements to make sure that the desired tested skills are covered by the output elements. This stage is where assignments can be tweaked to add or remove elements.

**Figure 2.**
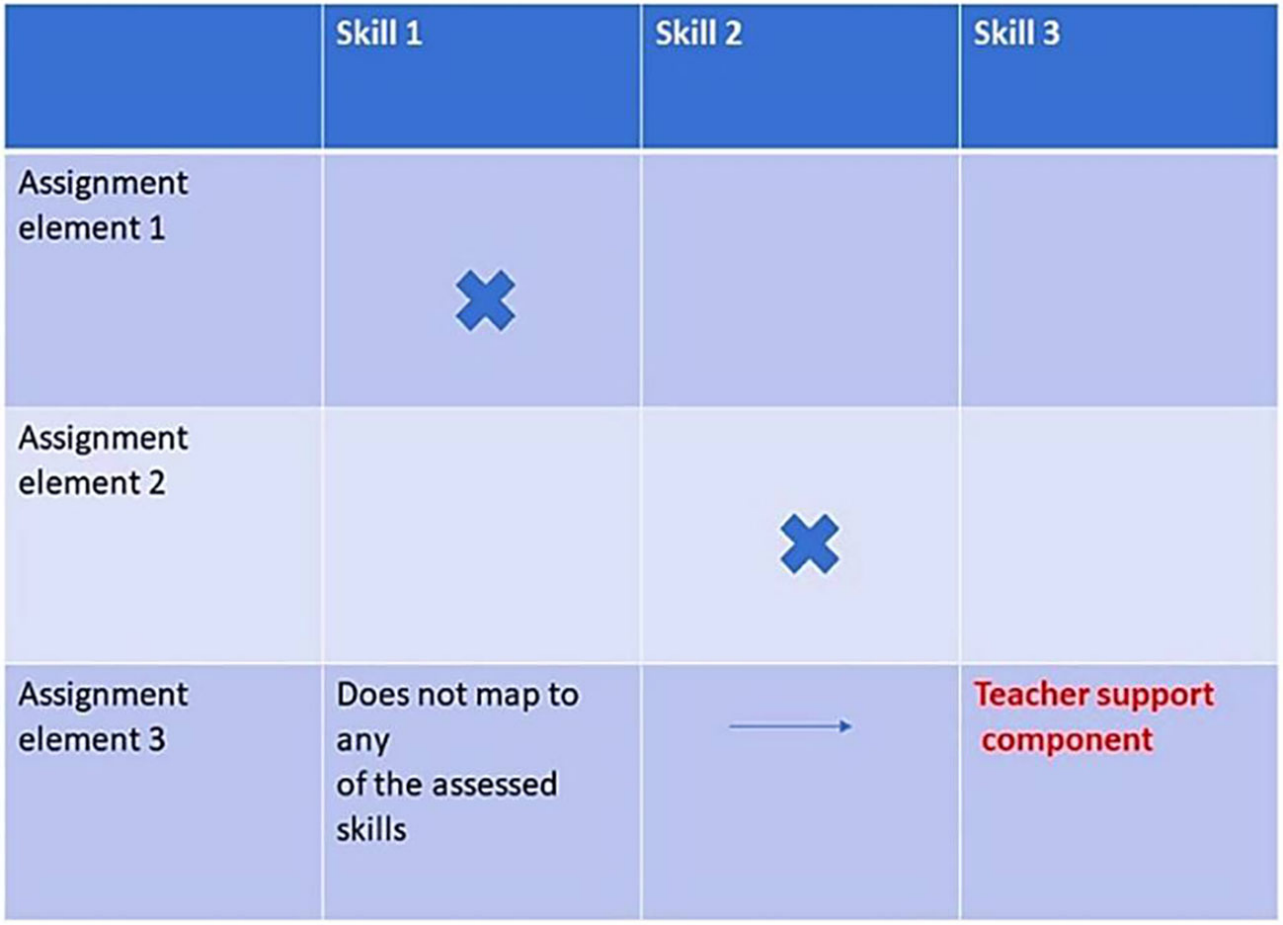
Matrix of assignment elements

An example of these elements is the elements needed to produce an educational video. These can be dissected in several ways but for example one of the elements would be the scientific content. Another element can be the video scenario and a third can be graphic design. Each of these elements should be mapped against the listed skills we are testing. If you cannot map it to a specific skill, then most probably it lies beyond the skill set needed to be assessed and so it becomes a potential area for support from teachers to their students during the assignment ( Figure 2).

##### Does it serve the social responsibility of the school?

Once the desired output is described evaluate this output in terms of its utility and its weight in social responsibility if the schools. Rate the projected product against its added value. Whenever the output can be identified as an asset or a product of extended value to others it is better.

##### Design Assignment instructions

Design written procedures that are simple and clear for the expected steps that organize implementation timeframe, team relationships, and advised steps.

##### Make sure the instructions are:

• Concise

Assignment Instructions should ideally be no more than one A4 paper long ( Rachul *et al.* 2020). Assignment instructions that are long cause students to feel overwhelmed and potion the assignment as a single response activity and encourage student plagiarism

• Detailed

Research has shown that the more detailed a writing assignment is, the better the student papers are in response to that assignment. Instructors can often help students write more effective papers by giving students written instructions about that assignment. Explicit descriptions of assignments on the syllabus or an “assignment sheet” tend to produce the best results. These instructions might make explicit the process or steps necessary to complete the assignment. Assignment sheets should detail:

• Purpose

Discuss the purpose of the assignment. Learners benefit from not just knowing what they are required to learn but also knowing why they are required to learn such knowledge, skills, or attitudes. Providing the context for an assignment will highlight its relevance in the course or program, but also its relevance for future clinical practice ( Emig, 1977).

##### Summary of the assignment explaining the overall task

The learning objectives to which the assignment ties back so students understand how the assignment fits into the course and their learning.

• How this assignment benefits student learning in terms of the course, their program, or their careers.
• Any assignments to which this assignment ties back or any assignments that will build off this assignment ( Marian.instructure.com, 2020)
• The kind of writing expected
• The scope of acceptable subject matter
• Length requirements
• Formatting requirements
• Documentation format
• The amount and type of research expected (if any)
• The responder’s role
• Deadlines for the first draft and its revision
• Providing questions or needed data in the assignment helps students get started. For instance, some questions can suggest a mode of organization to the students. Other questions might suggest a procedure to follow. The questions posed should require that students assert a thesis ( Cmsw.mit.edu, 2020)
• Add any reference the students will need to refer to: This should be related to the program specifications and preferably selected from faculty-approved references. The selection of specific and limited references helps the students easily reach their target goals. Make a clear point that allows for student independent search for references.
• Includes examples for clarification: Providing templates and the delivery of a demo presentation or video improves the quality of the output. Moreover, it unifies the output format.
• Includes information on who to contact for questions and detailed contact information. Adding contact information for students at the end of the assignment or an email to direct questions to gives students a sense of security and an opportunity to reach out for clarifications. The faculty assigned to answering student questions should be fully aware of the required assignment and the grading criteria. Consider holding a faculty orientation session before assigning student assignments to them.

##### Proofread

Make sure the assignment is read by any of your colleagues. Examine the assignment with reference to understandability, feasibility, logic. Revise the instructions to affirm that students are given enough space for creativity.

##### Plan an assessment guide

An assessment guide is a very important document for faculty to assess the student’s work. This document is an outline document that is attached to the assignment instruction document. This document is of maximum importance to ensure the reliability of the assessment tool.

##### Design Rubrics

Design a set of rubrics for each skill to be assessed. In the case of differentiating summative exams. Design multilevel achievement rubrics for each skill. In each level describe the level of achievement in measurable details.

##### Train faculty assessors

Make sure you train faculty on the use of rubrics in assessing process and output. Faculty who are using rubrics for the first time will need to be trained in a hands-on setting with mock assignments.

##### Step three: Implementation

The role of mentorship is irreplaceable for multiple reasons. Mentors do not only coach students to achieve the outcomes of the assignment but also provides the required context/structure for the work, monitor the timely achievement of assignment milestones and offer a role model to students in the means of teamwork, giving and receiving feedback as well as communication. Having a trained mentor will add value to the overall outcomes of the E-assignment and help students to develop professionalism attributes. Another tip might be adding two faculty members to mentor each group of students to support each other and provide more accessibility to students when they need advice.

##### Role of Faculty

The role of faculty during implementation is listed in Table 1.

**Table 1.** Role of faculty during the implementation of assignments

Role	Description
**Guidance**	• Providing guidance at the beginning of and throughout an assignment. • When appropriate, and particularly for new or complex concepts, outlining the potential learning strategies with learners can be helpful (Gagne et al., 1992; Al-Eraky, 2012).
**Resources**	• Offer resources addition to those offered in the assignment guidelines
**Directions**	• Provide clear expectations for an independent learning assignment will facilitate learning (Thomas et al., 2015; Hockings et al., 2018). • Identify areas of direction that can be given by faculty. These are the areas identified as outside the assessed skill set in the element blueprint. • Examples can be offered from similar assignments presented in preceding years
**Office hours**	• Designate office hours and make sure students are aware of the office hours of the contact person identified in the assignment
**Revise and approve the plan of action**	• The plan of action can be considered as one of the elements of the assignment.
**Quality Control**	• Ensure the integrity of process and methods by continuous follow up of the preapproved plan. • Ensure the quality of output in the pre-evaluation phase. • Quality assurance of the process and product before submission of the assignment. This can be done on a checklist generated from the instruction sheet. This checklist can be designed by the students as an element of their assignment. They can use this checklist within the process of self-evaluation, peer evaluation of faculty evaluation.
**Ensure the soundness of content**	• This is an excellent opportunity for faculty to teach professionalism to students to be open to their ideas, to teach them discipline, how to give and receive feedback, how to commit to the time frame previously planned by the team, and finally how to react to unexpected outcomes.

##### Role of student

The role of the student during implementation is listed in Table 2.

**Table 2.** Role of students during the implementation of assignments

Role	Description
**Inquiry- Check in with faculty in key stages of delivery**	• The student takes an active role in assignments to ask questions and reach out for required help from faculty • Students ate engaged and taking a more active role in their learning.
**Generate ideas-Test assumptions- Design an outline**	• The student takes charge of generating ideas and brainstorming them in groups until they come out with an assumption regarding the scope of the output.
**Set plans of action -Identify usable resources- Execute the plan**	• The plan is generated by the students after understanding the task and after having generated all the needed questions and elicited all the required information from the faculty. • The plan should contain a description of the product or output as well as a list of the tasks needed to be performed associated with a clear Gannt chart or timeline. • Tasks should be distributed among the group members provisionally and the whole plan should be approved by the faculty in charge. • Provide clear expectations for an independent learning assignment will facilitate learning (Thomas et al. 2015; Hockings et al. 2018).
**Self-evaluation- Peer evaluate in group assignments**	• Students are encouraged to evaluate themselves in the process and in the product as well as evaluate each other. • The timing of the evaluation should be integrated timely into the assignment instructions. • Peer assessment should be done at a phase in the execution where corrective action can be done. • It is preferred that it is conducted mid-way in the process in order to contribute to the student’s understanding of their contribution and how it can be improved.

### Step four: Assessment

Faculty approach assessment with a complete focus on two areas, both done in the rubric-guided process to ensure reliability.

#### Process assessment

Since faculty have been engaged throughout the planning and execution phase, they are capable of assessing the process that they witnessed and were a part of. Items to be assessed in the process include quality of the execution plan and student adherence to it, interpersonal communication within the group, etc.

#### Product assessment

The output is assessed against the pre-set rubrics. Assessment is done to grade or rank students based on items including quality of product and adherence to the guidelines, creativity, utility, and replicability.

Adherence to the rubrics established a near to reliable process that can be effective for summative assessment.

### Step five: Evaluation

Despite having the evaluation part as the last step, but it needs to be planned for at the early stages of designing the assignments. Evaluation equals organizational learning. Where all stakeholders can see tangible outcomes of the assignments and can learn from the experience of students and staff members while conducting these assignments that are usable and usable by others. Only through good evaluation, the next cycles of E-assignments will include better plans, more innovative approaches, and maybe more involvement of students and community members.

#### Decide upon level(s) of evaluation (Kirkpatrick)

Evaluating the assignments at multiple levels (according to Kirkpatrick’s model) provides planners and stakeholders with a comprehensive evaluation that helps further planning. The satisfaction of different parties is important and easy to evaluate including students, staff members, and other stakeholders. As acceptability of assignments is key to their success. Rubrics will help evaluate the learning component from the assignments which is another crucial outcome of the whole process. The behaviours of participating students should also be evaluated using tools that evaluate professionalism and other various competencies such as collaboration, communication, and community awareness. A suggested tool is the 30 degrees evaluation (AKA Multisource feedback) to let all involved individuals evaluate their peers. At last, comparing the students’ overall performance before and after the E-assignment might be another method to evaluate the impact of such a process.

#### Develop data collection tools (forms)

The planning team should pay attention to the development of data collection tools once they agree on the evaluation framework. Using validated tools for data collection is a possibility and saves a lot of the team’s effort and time. However, innovative assignments might require the development of authentic tools that will need validation by medical education experts.

The team will also need to consider not to overwhelm users with multiple forms. The role is always to select a manageable number of indicators while planning. Select the essential and not interesting indicators. Using electronic forms for data collection will provide better access to various stakeholders with also initial analysis that can be done.

#### Analyse results

A simple and representative analysis of the results will be an outcome of a well-planned design of the evaluation that is most importantly has clear objectives. Some very important outcomes from our perspective are acceptability of the assignment, evidence of learning among students, and the development of the students’ personalities and behaviours. Proper and deliberate analysis of results will defiantly make the next step easier and specific to serve the process of E-assignments.

#### Discuss results with stakeholders

Involving various stakeholders is an essential step to ensure not only more development and improvement of E-assignments but also to sustain them as a norm in health-professions education institutes and promote the acceptability of this method among other less-involved staff members. Stakeholders include decision-makers, staff members from the same school, staff, members from other schools who are partners, or potential partners in interdisciplinary assignments, students, and community members.

#### Decide on further planning and implementation for better outcomes

Considering the feedback from various stakeholders and evaluation results, discussions regarding further plans and next steps should take place to design the E-assignments for the next cycle. Learning from lessons and realize that we always have an opportunity for improvement are important norms for team planning and implementing E-assignments to consider.

#### Disseminate results

Part of the academic scholarship of such experience should include dissemination of the results of such a useful experience. This dissemination can be performed at the local level where students themselves can put their work on posters or E-posters and have the chance to display them to peers and staff in some event. Publication of the experience will also help expand on the use of such valuable learning and assessment tools.

#### Replicate experience

After proper dissemination, multiple parties can be inspired by such a unique, creative, and active learning tool. This will include the wider application of E-assignment to cover other curricular areas or other phases in the medical school as well as replication of the successful experience in other health professions or even non-health professions institutes.

## Case Study

An integrated assignment was held for 3rd-year medical students. This started with a needs assessment done through a set of questionnaires administered to students. The questionnaires included both quantitative (5-Likert scale questions) and qualitative data (open-ended questions). Priorities identified were discussed in a focus group. Rubrics for assignment grading were designed in a series of faculty meetings that resulted in an approved assignment guide containing the instructions and the rubrics that were announced to students before the start of the assignment. A plan was developed with students to build a question bank.

Training sessions were held for the staff members, then the students over Zoom on how to develop an item for a question bank. The scope of questions was identified for students guided by the integrated lecture schedule. Templates were developed and handed to students. Students were assigned specific integrated lectures to study and the task was to develop a case scenario item for each lecture. Thus, the primary outcome of acquiring deep understanding, develop analytical thinking, together with the clinical application are ensured.

Items are screened for face validity revised by mentors and those accepted had to pass through a three-student committee for peer review and comments and Cases were submitted to the required formatting process to be published to the students’ Question bank. As a result of this assignment, we ended up with a seven hundred-item bank completely revised and validated and ready to be added to the faculty item bank. The authors and reviewers were affiliated in the section they have created.

Online quiz competitions were held using the students’ Q bank items to maximize student engagement. Item analysis for post-validation was performed. The whole process was evaluated by analysing student responses to the questionnaire and opinions expressed in a focus group.

The students enjoyed the experience as was apparent from the low percentage of student drop out from the activity (4.7%) and the comments that were seen in the focus group follow up call that was held to collect student opinions and satisfaction level as indicated from what they expressed during the focus group:

“I wish this technique could be applied in all years”

“It was an extremely fruitful experience and it is a sincere pleasure working with this competent team. Next time, the teams should be more organised. Better communication between the organisers, writers, and reviewers. Starting up an online upload of the questions is a great step as well.”

“I found the experience very satisfying and didn’t find any problem concerning my part”.

“I got a good experience in this workshop. It’s well organized and the instructor was very encouraging and cooperating.”

I don’t think that this work is over. And I believe we should continue working to increase and improve our question bank so that it could withhold more topics and not necessarily only the topics predetermined by our Faculty. We could also and hopefully do such question banks for other fields too.

Other ideas for multidisciplinary assignment-projects can even cover even clinical skills and competencies. Proposed examples for this are creating virtual patients’ videos ( Berman *et al.,* 2016 and Consorti *et al.,* 2012). This can be used as an assignment for assessing algorithm reasoning among students.

## Take Home Messages

An integrated competency-based assignment can be well-tailored to an enthusiastic project, not only to provide a fair assessment but also, to create a usable product. Students’ engagement in the needs assessment, design, plan, implementation, and evaluation of the end product maximize the outputs to unexpected horizons. In addition to ensuring acquiring the intended skills and competencies.

## Notes On Contributors

**Hebat Allah A. Amin:** MSc, MD, AICPD, FAIMER fellow 2020. She is a lecturer of Histopathology, the Academic Co-chair of the Steering Committee for the MBBCh program, phase I coordinator, Head of the E-Learning Committee, and member in the exam Committee and the medical education unit, Faculty of Medicine, Helwan University (FMHU). ORCID ID: https://orcid.org/0000-0003-3311-4840

**Mohamed Hany K. Shehata:** MSc, MD, MHPE, FAIMER Fellow. He is a Professor of Family Medicine - AGU. Faculty in EMR Regional FAIMER Institute. He founded the Medical Education Unit at Helwan University. Worked as an educational consultant in the Egyptian Fellowship. In Suez Canal University he led the school’s teams of field training, Clinical teaching, and OSCE.ORCID ID: https://orcid.org/0000-0001-7069-9329

**Samar A. Ahmed:** Medical Doctorate, MHPE, FAIMER Fellow, UNESCO TOT, Full professor in Forensic Medicine Ain Shams University, Director of ASU-MENA-FRI. She has a wide experience in project management and proposal writing after being a part of the Ministry of Higher Education EU project team for quite some time. She held many educational positions as a director of the quality assurance unit and the Director of the education development unit in more than one university. ORCID ID: https://orcid.org/0000-0001-8119-9258

## Declarations

The author has declared that there are no conflicts of interest.

## Ethics Statement

Faculty of Medicine, Helwan University, Research Ethics Committee for Human & Animal Research (FMHU-REC) has approved the project entitled ‘Medical Students’ Contribution to Curriculum Reformation’, REC no 24/2020. The included case-study is the phase I implementation. FMHU-REC is organised and operated according to the Declaration of Helsinki.

## External Funding

This article has not had any External Funding
