# Supplementary material for: Step-by-step Guide to Create Competency-Based Assignments as an Alternative for Traditional Summative Assessment
Source: MedEdPublish (2016). 2020 Jun 8;9:120. [Version 1] doi: 10.15694/mep.2020.000120.1 (PMC10702678; doi:10.15694/mep.2020.000120.1)
Supplement: Peer review history

# Reviewer report: Reviewer response for version 1

**DOI:** 10.21956/mep.20072.r30857

**Published:** 2020-06-23

**Authors:** Keith Wilson

**Affiliations:** 1 Dalhousie University

This review has been migrated. The reviewer awarded 4 stars out of 5 The authors of this paper help the reader explore a roadmap to developing innovative assessments in an era of COVID-19. They rightly argue that we are exploring the exchange of formative and summative approaches to assessment.They provide a case example at the end of the article although I do feel this may better be served up front to provide context. Alternatively, as they elaborate on their suggested steps, elements of the case example could be woven in to give more ideas to the reader. The article has so many good elements/learning points that it may have been helpful to simplify down some of the messages – it could be turned into a new model of thinking about assessment. My worry is that some of the suggestions may be diluted down in the list. One of the most practical sections of the article is in the section on “Questions” – it provides some really great advice on how educators can build an innovative assessment. I feel that there are a number of areas where additional references would be beneficial for the reader. I would be happy to share these with the authors if interested. Overall this is a very interesting and timely article on how we can approach creative assessments in light of COVID-19. However, the real impact is how this approach can be realised beyond our restrictions during COVID-19. Anyone interested in assessment of students should read this article as it contains a number of practical tips.

Reviewer Expertise:

NA

No decision status is available

# Reviewer report: Reviewer response for version 1

**DOI:** 10.21956/mep.20072.r30847

**Published:** 2020-06-11

**Authors:** Gehan Sadek

**Affiliations:** 1 Menoufia Faculty of Medicine

This review has been migrated. The reviewer awarded 5 stars out of 5 Professional beneficial well-structured work. I got many new ideas from it. Can be very useful in the current situation regarding COVID 19 pandemic. Thanks for this work

Reviewer Expertise:

NA

No decision status is available

# Reviewer report: Reviewer response for version 1

**DOI:** 10.21956/mep.20072.r30852

**Published:** 2020-06-11

**Authors:** Anne D Souza

**Affiliations:** 1 Kasturba Medical College, Manipal Academy of Higher Education, Manipal, India

This review has been migrated. The reviewer awarded 4 stars out of 5 I enjoyed reading this article. Especially the description of the case study is quite impressive.The mapping of skills for each assignment could be a highly recommendable approach. The guidelines provided are precise and give a bird’s eye view on how an online assignment needs to be designed. The summary describes the entire process thoroughly.My suggestion is a flow diagram describing the entire process of creating e-assignments that could reinforce the summary.Thank you for this innovative and informative work.

Reviewer Expertise:

NA

No decision status is available

# Reviewer report: Reviewer response for version 1

**DOI:** 10.21956/mep.20072.r30864

**Published:** 2020-06-10

**Authors:** Susmita Reddy Karri

**Affiliations:** 1 Topiwala national medical college and BYL nair charitable hospital, mumbai. india

This review has been migrated. The reviewer awarded 5 stars out of 5 Great work. A very thorough and well structured work.

Reviewer Expertise:

NA

No decision status is available

# Reviewer report: Reviewer response for version 1

**DOI:** 10.21956/mep.20072.r30851

**Published:** 2020-06-10

**Authors:** Felix Silwimba

**Affiliations:** 1 University of Lusaka

This review has been migrated. The reviewer awarded 5 stars out of 5 Well detailed and clearly shared knowledge on e-assessments. I have personally picked a number of good ideas.

Reviewer Expertise:

NA

No decision status is available

# Reviewer report: Reviewer response for version 1

**DOI:** 10.21956/mep.20072.r30846

**Published:** 2020-06-09

**Authors:** mariam asaad amin ibrahim

**Affiliations:** 1 ain shams faculty of medicine

This review has been migrated. The reviewer awarded 5 stars out of 5 Very innovative and professionalThank you for the excellent guide

Reviewer Expertise:

NA

No decision status is available

# Reviewer report: Reviewer response for version 1

**DOI:** 10.21956/mep.20072.r30862

**Published:** 2020-06-09

**Authors:** Mustafa.H Shahin

**Affiliations:** 1 Helwan University

This review has been migrated. The reviewer awarded 5 stars out of 5 It really was a great experince to join and work in a great team . I hope to make more and more achievments in the near future with this enthusiastic and cooparative partners.

Reviewer Expertise:

NA

No decision status is available

# Reviewer report: Reviewer response for version 1

**DOI:** 10.21956/mep.20072.r30849

**Published:** 2020-06-09

**Authors:** dalia gaber

**Affiliations:** 1 faculty of medicine, helwan university

This review has been migrated. The reviewer awarded 5 stars out of 5 Thank you for establishing such amazing work that can be a guide for medical educators as an innovative assessment tool.

Reviewer Expertise:

NA

No decision status is available

# Reviewer report: Reviewer response for version 1

**DOI:** 10.21956/mep.20072.r30856

**Published:** 2020-06-08

**Authors:** Iman Salem

**Affiliations:** 1 KAU

This review has been migrated. The reviewer awarded 5 stars out of 5 It is an amazing idea and a great workThanks for your efforts

Reviewer Expertise:

NA

No decision status is available

# Reviewer report: Reviewer response for version 1

**DOI:** 10.21956/mep.20072.r30855

**Published:** 2020-06-08

**Authors:** Nagwa Hegazy

**Affiliations:** 1 Menoufia university

This review has been migrated. The reviewer awarded 4 stars out of 5 Thanks for this authentic detailed work.It suits the current era. I enjoyed reading the suggested model of E-Assignments levels and the matrix for the assignment elements. That was really descriptive. I believe that combining it with Miller pyramid could be a good step. My question for the authors; isn't product assessment part of the evaluation? The presence of the case study had shown the applicability of the proposed model.Thanks again for this great work

Reviewer Expertise:

NA

No decision status is available

# Reviewer report: Reviewer response for version 1

**DOI:** 10.21956/mep.20072.r30844

**Published:** 2020-06-08

**Authors:** Zeinab Kasemy

**Affiliations:** 1 Menoufia faculty of medicine

This review has been migrated. The reviewer awarded 5 stars out of 5 Nice work

Reviewer Expertise:

NA

No decision status is available

# Reviewer report: Reviewer response for version 1

**DOI:** 10.21956/mep.20072.r30860

**Published:** 2020-06-08

**Authors:** Haidy Khalil

**Affiliations:** 1 Faculty of Medicine / Helwan University

This review has been migrated. The reviewer awarded 5 stars out of 5 Very fruitful and to the point, Great work.

Reviewer Expertise:

NA

No decision status is available

# Reviewer report: Reviewer response for version 1

**DOI:** 10.21956/mep.20072.r30861

**Published:** 2020-06-08

**Authors:** Amany Shabaiek

**Affiliations:** 1 Forensic medicine authority

This review has been migrated. The reviewer awarded 5 stars out of 5 Meticulous innovative work and great effort.. From a professional team.. God blesses you

Reviewer Expertise:

NA

No decision status is available

# Reviewer report: Reviewer response for version 1

**DOI:** 10.21956/mep.20072.r30859

**Published:** 2020-06-08

**Authors:** Aya Elmahs

**Affiliations:** 1 Helwan faculty of medicine

This review has been migrated. The reviewer awarded 5 stars out of 5 Great work as usual Dr Heba and Dr Mohamed.. Being a part of Helwan faculty of Medicine, I can see how those assignments develop students' skills.. Hope this will be a part of routine practice in Helwan after Covid elemenation..

Reviewer Expertise:

NA

No decision status is available

# Reviewer report: Reviewer response for version 1

**DOI:** 10.21956/mep.20072.r30843

**Published:** 2020-06-08

**Authors:** Nagwa Kostandy Kalleny Nasrallah Nasrallah

**Affiliations:** 1 Faculty of Medicice Ain Shams University

This review has been migrated. The reviewer awarded 5 stars out of 5 A great professional very helpful work. Keep it up.

Reviewer Expertise:

NA

No decision status is available

# Reviewer report: Reviewer response for version 1

**DOI:** 10.21956/mep.20072.r30845

**Published:** 2020-06-08

**Authors:** Dalia Abouelfadl

**Affiliations:** 1 National

This review has been migrated. The reviewer awarded 5 stars out of 5 Great work as usual Dr HebaVery beneficial in times of pandemics like these days.Keep going

Reviewer Expertise:

NA

No decision status is available

# Reviewer report: Reviewer response for version 1

**DOI:** 10.21956/mep.20072.r30865

**Published:** 2020-06-08

**Authors:** sally saied

**Affiliations:** 1 egyptian medicolegal authority

This review has been migrated. The reviewer awarded 5 stars out of 5 Great job and amazing effort, meticulous results. very professional And useful work

Reviewer Expertise:

NA

No decision status is available

# Reviewer report: Reviewer response for version 1

**DOI:** 10.21956/mep.20072.r30863

**Published:** 2020-06-08

**Authors:** Shaimaa Bahr

**Affiliations:** 1 Helwan University

This review has been migrated. The reviewer awarded 5 stars out of 5 Very informative,beneficial and applicable! Great work doctors.

Reviewer Expertise:

NA

No decision status is available

# Reviewer report: Reviewer response for version 1

**DOI:** 10.21956/mep.20072.r30854

**Published:** 2020-06-08

**Authors:** Safa Alkalash

**Affiliations:** 1 Menoufia univeristy

This review has been migrated. The reviewer awarded 4 stars out of 5 Excellent and great work dear professors.

Reviewer Expertise:

NA

No decision status is available

# Reviewer report: Reviewer response for version 1

**DOI:** 10.21956/mep.20072.r30858

**Published:** 2020-06-08

**Authors:** Jihan Ahmed Elwahab

**Affiliations:** 1 Environmental sciences and research institution

This review has been migrated. The reviewer awarded 5 stars out of 5 A strong paper based on current situation and establishing quick response to educational proplems. And great effort

Reviewer Expertise:

NA

No decision status is available

# Reviewer report: Reviewer response for version 1

**DOI:** 10.21956/mep.20072.r30853

**Published:** 2020-06-08

**Authors:** Sherin Naguib

**Affiliations:** 1 Egyptian Forensic medicine authority

This review has been migrated. The reviewer awarded 5 stars out of 5 Great and professional job, hard effort, and amazing results

Reviewer Expertise:

NA

No decision status is available

# Reviewer report: Reviewer response for version 1

**DOI:** 10.21956/mep.20072.r30848

**Published:** 2020-06-08

**Authors:** Lamyaa El Hassan

**Affiliations:** 1 Faculty of Medicine. Jazan University

This review has been migrated. The reviewer awarded 5 stars out of 5 Excellent. Very informative and useful.

Reviewer Expertise:

NA

No decision status is available

# Reviewer report: Reviewer response for version 1

**DOI:** 10.21956/mep.20072.r30850

**Published:** 2020-06-08

**Authors:** Nayera Mostafa

**Affiliations:** 1 Faculty of medicine, Ain Shams University

This review has been migrated. The reviewer awarded 4 stars out of 5 Great and beneficial work. Keep it up!

Reviewer Expertise:

NA

No decision status is available
